# A brief review of the current status of pig islet xenotransplantation

**DOI:** 10.3389/fimmu.2024.1366530

**Published:** 2024-02-23

**Authors:** David K. C. Cooper, Lisha Mou, Rita Bottino

**Affiliations:** ^1^ Center for Transplantation Sciences, Massachusetts General Hospital/Harvard Medical School, Boston, MA, United States; ^2^ Institute of Translational Medicine, The First Affiliated Hospital of Shenzhen University, Shenzhen Second People’s Hospital, Shenzhen, Guangdong, China; ^3^ MetaLife Center, Shenzhen Institute of Translational Medicine, Shenzhen, Guangdong, China; ^4^ Imagine Islet Center, Imagine Pharma, Pittsburgh, PA, United States

**Keywords:** diabetes, islets, pancreatic, nonhuman primates, pig, genetically-engineered, xenotransplantation

## Abstract

An estimated 1.5 million Americans suffer from Type I diabetes mellitus, and its incidence is increasing worldwide. Islet allotransplantation offers a treatment, but the availability of deceased human donor pancreases is limited. The transplantation of islets from gene-edited pigs, if successful, would resolve this problem. Pigs are now available in which the expression of the three known xenoantigens against which humans have natural (preformed) antibodies has been deleted, and in which several human ‘protective’ genes have been introduced. The transplantation of neonatal pig islets has some advantages over that of adult pig islets. Transplantation into the portal vein of the recipient results in loss of many islets from the instant blood-mediated inflammatory reaction (IBMIR) and so the search for an alternative site continues. The adaptive immune response can be largely suppressed by an immunosuppressive regimen based on blockade of the CD40/CD154 T cell co-stimulation pathway, whereas conventional therapy (e.g., based on tacrolimus) is less successful. We suggest that, despite the need for effective immunosuppressive therapy, the transplantation of ‘free’ islets will prove more successful than that of encapsulated islets. There are data to suggest that, in the absence of rejection, the function of pig islets, though less efficient than human islets, will be sufficient to maintain normoglycemia in diabetic recipients. Pig islets transplanted into immunosuppressed nonhuman primates have maintained normoglycemia for periods extending more than two years, illustrating the potential of this novel form of therapy.

## Introduction

Type 1 diabetes (T1D) is an autoimmune disease characterized by insulin-secreting β cell destruction by CD4^+^ and CD8^+^ T cells, resulting in insulin deficiency and hyperglycemia. Genetic susceptibility plays a role in the development of T1D, which is associated in part with certain human leukocyte antigens (HLA) (1). Conventional treatment of T1D includes exogenous insulin therapy, which helps reduce hyperglycemia. However, in patients with unstable (‘brittle’) diabetes, it is difficult to prevent life-threatening hypoglycemia or hyperglycemia, as well as late complications, e.g., retinopathy, nephropathy, vascular disease (2). Islet allotransplantation is viewed as an efficient therapy for T1D.

Studies have demonstrated that islet transplantation can significantly reduce, or eliminate, the need for daily insulin injections, marking a pivotal shift in T1D management (3). Furthermore, the enhanced quality of life, coupled with a notable reduction in diabetes-related complications, underscores the transformative potential of islet transplantation (4). By integrating detailed outcomes from relevant research, this introduction aims to illustrate the broader implications of islet transplantation, not only as a mechanism for blood sugar regulation but also to provide new solutions for the treatment of patients with T1D.

However, the shortage of pancreases from deceased human donors poses a problem of increasing need for another source of islets, which may be met by gene-edited pigs (5–7).

Indeed, xenotransplantation has immense potential for the treatment of numerous disorders and will prove to be the next great medical revolution (8). Pancreatic islet transplantation will benefit greatly from an unlimited number of gene-edited pigs. With the potential advantages of neonatal islets (see below), the transplantation of neonatal islet-like cell clusters (NICC), which will never be available in sufficient numbers from deceased human neonates, will become possible.

As there are an estimated 1.5 million patients with T1D and perhaps 30 million with type 2 diabetes in the USA alone, the number of islet transplants carried out worldwide will increase exponentially. The islet grafts will control the patient’s blood glucose for long periods of time (if not permanently) without the need for daily insulin injections. Because of the ready availability of the islet-source pigs, islet re-transplantation will be possible whenever required and will be a relatively simple procedure.

## History of islet xenotransplantation

Insulin deficiency can be overcome by transplanting pancreatic allo-islets (9). Early attempts, none of which succeeded, were reported in the late 19^th^ and early 20^th^ centuries (6). Novel insights in pancreatic islet cell biology, the development of improved methods of islet isolation (10), and the introduction of an automated approach for isolating islets from human pancreases were major steps forward (11).

In regard to islet xenotransplantation, the pig represents the most probable source of islets for various reasons (**Table 1**) (5). The sequence of porcine insulin differs by only a single amino acid from that of human insulin and, moreover, porcine insulin was administered to treat diabetes successfully for nearly a century before the introduction of recombinant human insulin (12).

**Table 1:** Advantages and disadvantages of the pig as a potential source of organs and cells for humans, in contrast to the baboon in this role.

	Pig	Baboon
**Availability**	Unlimited	Limited
**Breeding potential**	Good	Poor
**Period to reproductive maturity**	4-8 months	3-5 years
**Length of pregnancy**	114 + 2 days	173-193 days
**Number of offspring**	5-12	1-2
**Growth**	Rapid (adult human size by 6 months) *^a^*	Slow (9 years to reach maximum size)
**Size of adult organs**	Adequate	Inadequate *^b^*
**Cost of maintenance**	Significantly lower	High
**Anatomical similarity to humans**	Close	Close
**Physiological similarity to humans**	Moderately close	Close
**Immune system in relation to humans**	Distant	Close
**Knowledge of tissue typing**	Considerable (in selected herds)	Limited
**Necessity for blood type compatibility with humans**	Probably unimportant	Important
**Experience with genetic engineering**	Considerable	None
**Risk of transfer of infection (xenozoonosis)**	Low	High
**Availability of designated pathogen-free animals**	Yes	No
**Public opinion**	More in favor	Mixed

^a^Breeds of miniature swine vary greatly in size.

^b^The size of certain organs, e.g., the heart, would be inadequate for transplantation into adult humans.

In the realm of islet xenotransplantation, porcine C-peptide measurements serve as a critical marker for evaluating the survival and functionality of transplanted pig islets in human recipients. This test, measuring the level of C-peptide, a byproduct of insulin production, provides insights into the pancreatic beta cells’ ability to produce insulin post-transplantation. Notable studies include Groth et al. (13), which marked the first human islet xenotransplantation attempt, though without significant improvement in glycemic control. The study by Elliott et al. (14) demonstrates the viability of pig islet xenotransplantation through C-peptide tests. The transplantation of neonatal pig islets into diabetic subjects showed a reduction in insulin dosage and an increase in serum pig C-peptide for up to two years, indicating sustained graft function. This evidence supports the potential of pig islets to survive and function in humans, offering a promising avenue for diabetes treatment by reducing insulin dependency.

Valdes-Gonzalez et al. (15) observed a reduction in insulin needs and improvements in HbA1c over time, indicating sustained functionality of transplanted islets. Wang et al. (16) and subsequent trials (17, 18) further supported these findings, demonstrating the potential of porcine islets to ameliorate diabetes management, despite varying degrees of success and the absence of long-term insulin independence in all cases.

## The optimal age of the pig as a source of islets

The ideal age of the islet-source pig has been discussed for many years. Pigs can be divided into three age groups – fetal, neonatal (approximately <14 days-old), and adult (>12 weeks-old (**Table 2**). As fetal pig islets are not currently being considered for xenotransplantation (because of limited β-cell yield and delayed production of insulin), the choice is between adult or neonatal pigs. There are advantages and disadvantages to both (19, 20).

**Table 2:** Advantages and disadvantages of neonatal and adult pig islets for clinical xenotransplantation.

	Neonatal	Adult
**Isolation procedure**	Simple	Difficult
**Cost of islet isolation**	Low	High
**Islet yield/pancreas (IEQs)**	25,000-50,000	200,000-500,000
**Beta cells (% of islet cells)**	25%	>70%
**Insulin production**	May be delayed	Immediate
**Proliferation *in vivo***	Yes	Little/none
**Tumorigenicity**	Low	None
**Risk of pathogen transmission**	Low	Low
**Cost of housing pig until islets utilized**	Low	High

**Figure 1:**
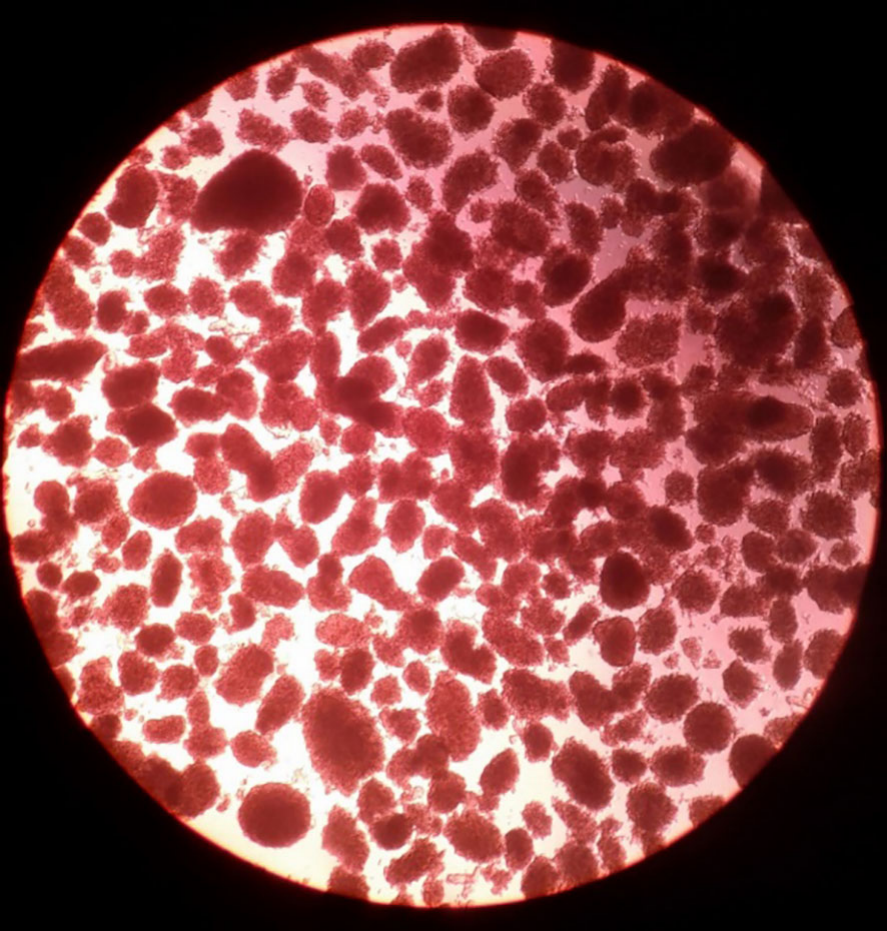
Adult pig islets after isolation. Adult pig islets stained in red with dithizone after isolation and purification (magnification 40x). (Reproduced with permission from 6).

Adult pig pancreases provide more fully-differentiated islets that are thus able to secrete insulin immediately after transplantation (**Figure 1**) (6). One adult pig pancreas may yield a sufficient number of islets to control diabetes after transplantation into a diabetic patient weighing 60kg (21). However, limitations are (i) the high cost of maintaining the pig until of adequate size (at approximately 6 months of age), (ii) the difficulty and high cost of islet isolation, and (iii) poor proliferation of the islets after transplantation (22) (**Table 2**). Adult sows (female pigs) that have delivered more than two litters of piglets (i.e., retired breeders, usually >2 years-old and weighing >200kg), may have advantages over young adult pigs as sources of islets by providing a greater yield of high-quality islets (20). However, the cost of maintaining them for two years would be considerable.

The advantages of neonatal islets (i.e., NICC) include (i) low cost of maintaining the piglets before pancreatectomy (<2 weeks), (ii) much simpler and reproducible NICC isolation, (iii) lower isolation costs compared to adult pig islets (22), and (iv) considerable proliferation of islets after transplantation (**Table 2**) (23). They may also be less susceptible to anoxic injury post-transplant. However, they have limitations – (i) a greater number is required to provide sufficient islets for a single adult human recipient, and (ii) they must be cultured to mature and re-aggregate before transplantation. Neonatal pigs can yield approximately 25,000-30,000 islets per donor pancreas. However, considering that a patient may require 10,000-20,000 porcine islet equivalents (IE)/kg for effective treatment, a 70kg patient may need as many as 25 or more piglet donors (10,000IE/kg x 70kg) (24). Nevertheless, if diabetes can be efficiently treated, this approach is justified (25).

Neonatal pigs are currently considered by many researchers as the favored age for obtaining islets for clinical use (26). The much greater costs of maintaining the pig until adulthood and of adult islet isolation may eventually prove decisive in favor of neonatal pigs as sources of islets for commercial clinical transplantation.

## The optimal site for pig islet xenotransplantation

This is another topic that has been debated for many years. The portal vein/liver is presently the favored location for islet allotransplantation (9). Nevertheless, the liver is not an optimal site for islet engraftment (27). Intraportal islet infusion increases the risk of hemorrhage and portal vein thrombosis. Furthermore, oxygen tension in the portal vein is lower than in the pancreas, which may lead to islet cell apoptosis. Most importantly, the instant blood-mediated inflammatory reaction (IBMIR – see below) may reduce the number of surviving islets by 60% within the first few hours or days (28–32). Furthermore, due to the broad distribution, biopsies of the engrafted islets are challenging and graft retrieval impossible. Alternative sites therefore continue to be explored (**Table 3**) (5, 26, 27). Transplant sites tested include the omental pouch, striated muscle, renal subcapsular space, the gastrointestinal submucosal space, and bone marrow.

**Table 3:** Comparison of different sites for free (non-encapsulated) islet xenotransplantation*^a^*.

	Liver	Renal capsule	Spleen	Skin	Omentum	Gastric submucosal space	Pancreas	Muscle
**Efficacy of clinical trials**	Good	Poor	Not reported	Poor	Limited experience	Limited experience	Not reported	Limited experience
**Patient safety**	Safe	Safe	Safe	Safe	Safe	Safe	Possible pancreatitis	Safe
**Oxygen tension**	Low	Not reported	High	Low	Not reported	High	Not reported	Not reported
**Vasculature**	Rich	Poor	Not reported, but probably rich	Poor	Rich	Rich	Not reported	Rich
**Site of insulin released by the graft**	Liver	Not reported	Portal vein	Systemic circulation	Portal vein	Portal vein	Not reported	Systemic circulation
**Surgery**	Invasive, some complications	Invasive	Invasive	Non-invasive	Easy	Easy (endoscopy)	Difficult	Easy
**IBMIR**	Yes	Not reported	Yes	Not reported	Not reported	Not reported	Not reported	Not reported

^a^Table modified from (27).

Islet transplantation into the renal subcapsular space has shown some success in experimental models, but limited success has been reported in humans, possibly from ischemic injury associated with compression of the islets. Preclinical studies in which pig islets were successfully transplanted either under the kidney capsule of pig littermates or in an autologous setting (thus in the absence of an immune response), demonstrated islet survival and revascularization (33). The established composite islet-kidney was then transplanted into an immunosuppressed allogeneic recipient. In Major Histocompatibility Complex (MHC)-matched pigs, successful engraftment and immediate function of both the islets and kidney was reported. In a similar model, successful engraftment was also reported in an immunosuppressed nonhuman primate (NHP) model (34).

To ensure the clinical relevance of these studies, it would be essential to utilize a xenogeneic model. Now that the rejection of a pig kidney can largely be prevented (35–37; Kinoshita et al. 2024^1^), it is time to explore this approach again. The primary objective is to utilize the combined pig islet-kidney to effectively treat both renal failure and diabetes in individuals suffering from diabetic nephropathy. This would probably best be achieved by implanting pig NICC into identical piglet recipients (possibly littermates), with subsequent transplantation of the islet/kidney into the patient.

## Gene editing of the islet-source pig

Quite remarkably, adult wild-type (WT, i.e., genetically-unmodified) pig islets have functioned in anti-CD154mAb-based immunosuppressed diabetic NHPs for up to 965 days (38). However, gene-editing of the pig would almost certainly have been associated with equally good or even better results with less intensive immunosuppressive therapy. Gene editing includes (i) deletion of expression of the 3 known pig carbohydrate xenoantigens (**Table 4**), and/or (ii) the introduction of one or more human ‘protective’ transgenes, e.g., complement-regulatory, coagulation-regulatory, and anti-inflammatory (anti-apoptotic) (5, 39).

**Table 4:** Known carbohydrate xenoantigens expressed on pig cells.

Carbohydrate (Abbreviation)	Responsible enzyme	Gene-knockout pig
Galactose-α1,3-galactose (Gal)	α1,3-galactosyltransferase	GTKO
N-glycolylneuraminic acid (Neu5Gc)	CMAH	CMAH-KO
Sda	β-1,4N-acetylgalactosaminyltransferase	β4GalNT2-KO

CMAH, Cytidine monophosphate-N-acetylneuraminic acid hydroxylase (CMAH).

**Figure 2:**
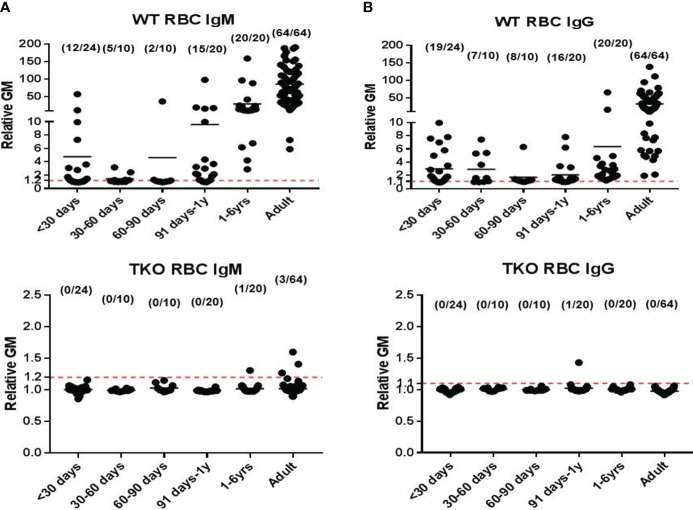
Human serum antibody binding to WT and TKO pig red blood cells (pRBCs). Correlation between human serum antibody binding to pig RBCs, by relative geometric mean [rGM]) and age. Human serum **(A)** IgM and **(B)** IgG antibody binding to wild-type (WT) pRBCs (top) and to Triple-knockout (TKO) pRBCs (bottom). The dotted lines indicate no IgM or IgG binding. (Note the great difference in the scale on the Y axis between A and B.) There is almost no anti-TKO pig antibody production during the first year of life and very low levels in adults compared to antibody against WT pig cells. (Reproduced with permission from 40).

**Figure 3:**
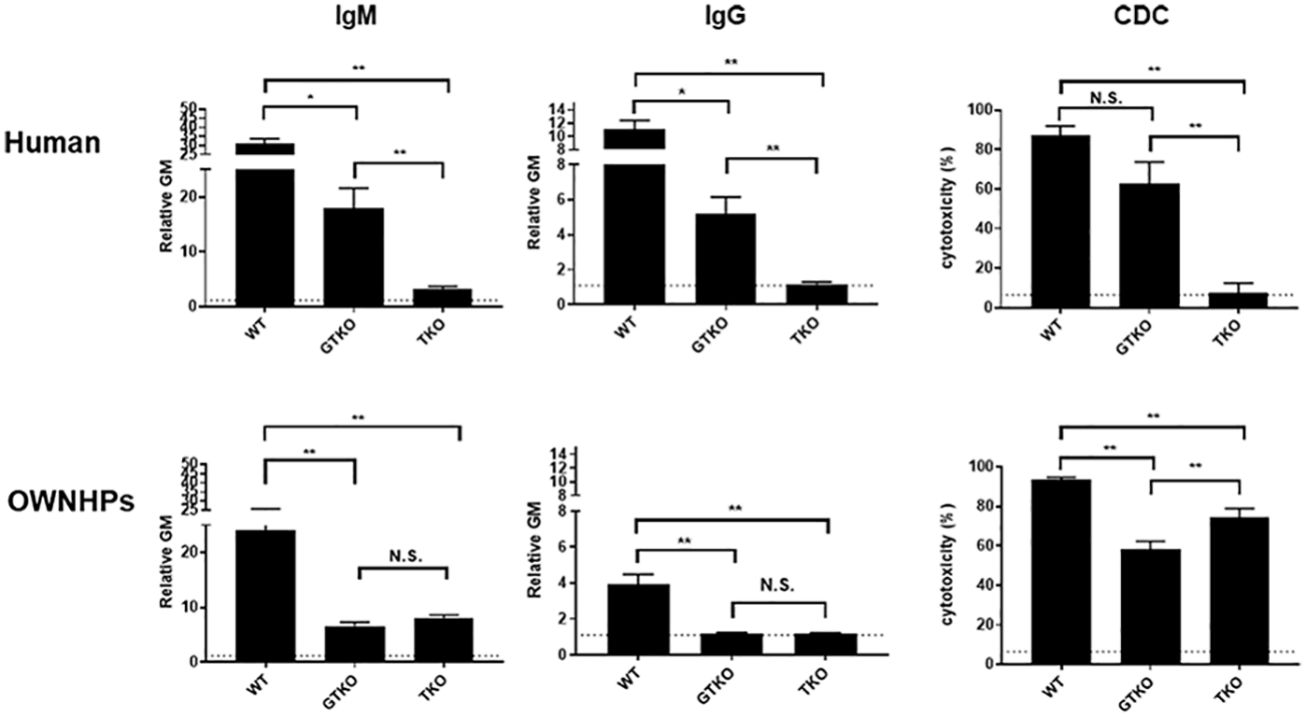
Human and Old World monkey serum antibody binding and cytotoxicity to WT, GTKO, and TKO pig peripheral blood mononuclear cells (PBMCs). Human (top) and Old World monkey (OWNHPs) (bottom) IgM (left) and IgG (middle) binding and complement-dependent cytotoxicity (CDC, at 25% serum concentration) (right) to WT, GTKO, and TKO pig PBMCs. Results are expressed as mean ± SEM. (*p<0.05, **p<0.01; N.S. = not significant). On the y axis, the dotted line represents cut-off value of binding (relative geometric mean [GM]: IgM 1.2, IgG 1.1), below which there is no binding. For CDC on the y axis, the dotted line represents cut-off value of cytotoxicity (6.4%), below which there is no cytotoxicity. (Note the difference in scale on the y axis between IgM and IgG.) Although there is reduced antibody binding and cytotoxicity to GTKO PBMCs in both humans and monkeys, there is an increase in antibody binding and cytotoxicity to TKO PBMCs in monkeys. (Reproduced with permission from 40).

Knockout of the genes for the 3 glycan xenoantigens (providing triple-knockout, [TKO] pigs) is generally considered the basis of the pigs that will be sources of organs and cells for clinical transplantation (**Figure 2**) (39). However, while TKO pig organs represent a significant advancement in xenotransplantation, the presence of pre-existing antibodies in Old World NHPs against these cells (**Figure 3**) (41) presents a complex challenge in pre-clinical studies, necessitating careful consideration and ongoing research to enhance compatibility and reduce immunological rejection.

**Figure 4:**
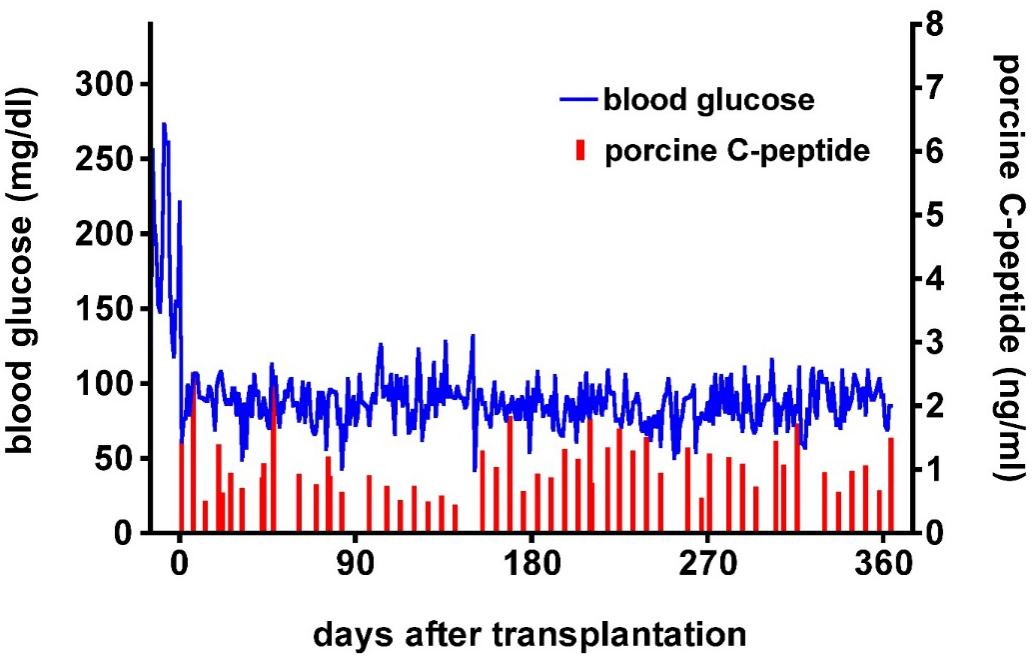
Post-transplant course of an immunosuppressed diabetic monkey following hCD46 pig islet transplantation. Blood glucose (blue) and pig C-peptide levels (red) in a streptozotocin-induced diabetic cynomolgus monkey before and after intraportal transplantation of islets from a pig expressing the human complement-regulatory protein, CD46. No exogenous insulin was administered after the transplant. The normoglycemic monkey was electively euthanized after 12 months. Day 0 = day of islet transplantation. (Reproduced with permission from 43).

There is evidence that the expression of protective human proteins adds to survival of pig organs or islets in NHPs. The adverse role of complement in pig islet xenotransplantation is well-known (42). The expression of one or more human complement-regulatory proteins (e.g., CD46, CD55, CD59) on the islets is therefore beneficial (43–45). In 2009, van der Windt et al. achieved insulin-independence in a diabetic monkey for >1 year by transplanting WT pig islets expressing a single human complement-regulatory protein, hCD46 (**Figure 4**) (43). More recently, Hawthorne and his colleagues achieved consistent long-term function of neonatal islets from GTKO pigs expressing human CD55 and CD59 in immunosuppressed baboons (46).

Expression of one or more human coagulation-regulatory proteins (e.g., thrombomodulin, endothelial cell protein C receptor [EPCR]), contributes resistance to IBMIR (47). The additional expression of a human anti-inflammatory gene (e.g., hemeoxygenase-1 [HO-1] or A20) and/or soluble human tumor necrosis factor receptor I IgG1-Fc provides some protection from the effects of inflammation (39, 48). Our group demonstrated modulation of IBMIR-mediated islet damage by employing multiple human transgenes that included complement and coagulation inhibitors. Despite reduced early islet damage, however, long-term improved outcome was not achieved (44).

There are further specific gene edits that can be made to the pig to modulate the cellular response to the islet graft, e.g., (i) insertion of a mutant (human) MHC class II transactivator gene which down-regulates swine leukocyte antigen (SLA) class II expression, (ii) deletion of expression of SLA class I (SLA class I-KO), or (iii) insertion of a CTLA4-Ig gene to induce local immunosuppression, (iv) expression of PD-L1, and (v) expression of HLA E and G (49–54).

## Immunosuppressive therapy

Gene edits designed to protect against innate immunity do not prevent the adaptive immune response (cellular rejection). Exogenous pharmacological immunosuppression is therefore required to modulate the immune response.

**Figure 5:**
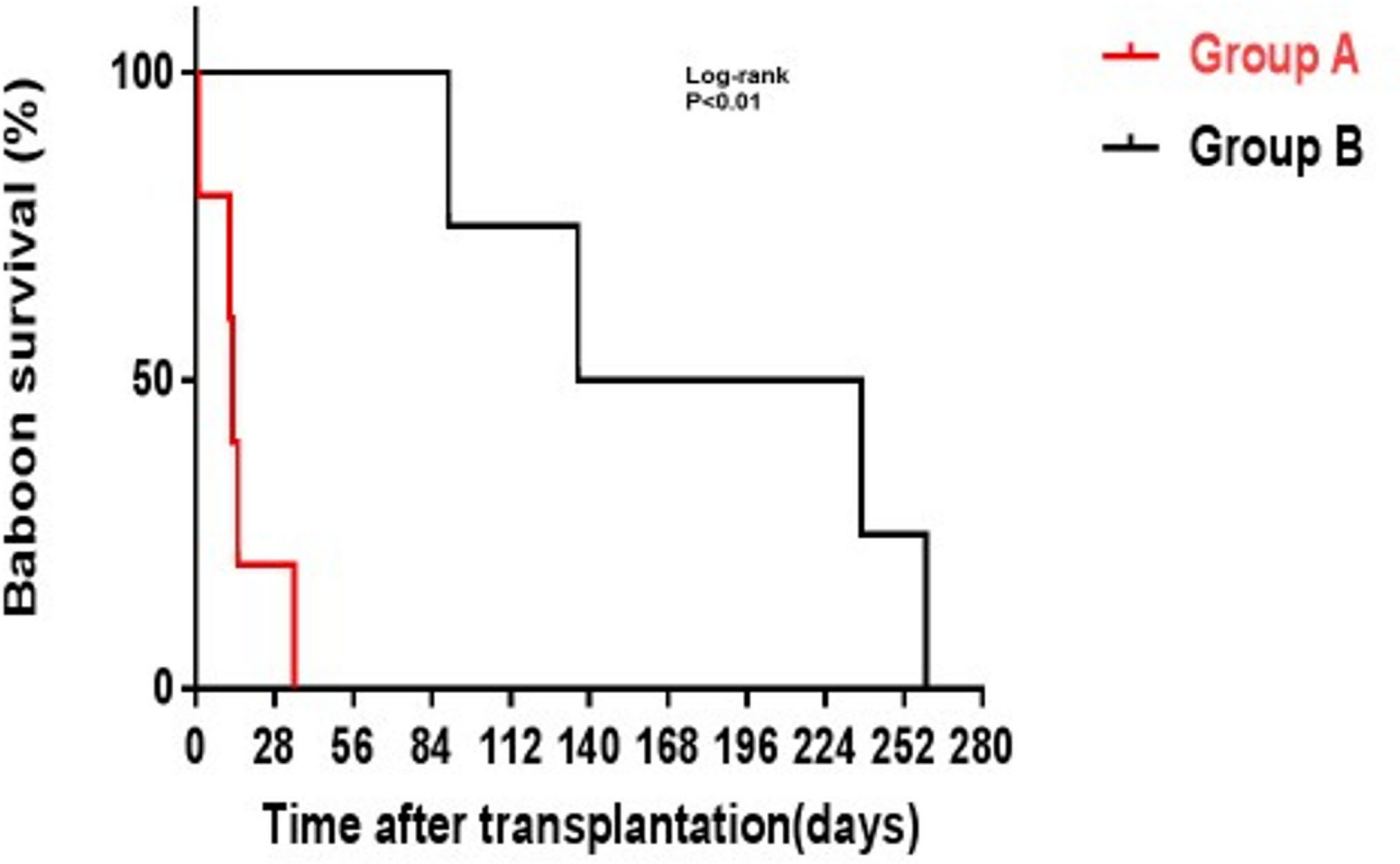
GTKO pig kidney survival in baboons receiving US FDA-approved immunosuppressive agents (Group A, in red) was much shorter than in those receiving an anti-CD40mAb-based regimen (Group B, in black). (Reproduced with permission from 56).

Buhler et al. were the first to demonstrate that conventional immunosuppressive therapy, e.g., tacrolimus-based, was inefficient in suppressing the adaptive immune response to a pig xenograft, but that blockade of the CD40/CD154 T cell co-stimulation pathway was much more successful (55). This observation has since been supported by numerous studies including several involving pig islet transplantation in NHPs (38, 43, 44, 56) (**Figure 5**). Some induction therapy (e.g., anti-thymocyte globulin, an anti-CD20mAb, and possibly transient inhibition of systemic complement activity) appears to be essential (**Table 5**) (57; Kinoshita et al.^1^). Anti-CD154 mAbs have proved more effective than anti-CD40 mAbs (38, 58), but were originally associated with thrombogenic complications (59), though these were *not* seen after pig islet transplantation (60). However, current modified anti-CD154 mAbs induce no thromboembolic complications in NHPs (36, 37; Kinoshita et al.^1^).

**Table 5:** Representative immunosuppressive and adjunctive regimen currently administered in our center to baboons with life-supporting TKO pig kidney grafts (which would be similar for TKO pig islet transplantation).

Agent	Dose (duration)
Induction	
Thymoglobulin (ATG)	5mg/kg i.v. (day -3) (to reduce the lymphocyte count to <500/mm^3^)
Anti-CD20mAb (rituximab)	10mg/kg i.v. (day -2)
C1-esterase inhibitor	17.5U/kg i.v. on days 0 and 2.
Maintenance	
Anti-CD154 mAb (Tonix-1500)	30mg/kg (days 0, 2, 7, 10, 14, and weekly)
Rapamycin	0.1-0.2mg/kg i.m./day (target trough 6-12 ng/ml) beginning on day -5.
Methylprednisolone	10mg/kg/d on day 0, tapering to 0.25 mg/kg/d by day 7.
Adjunctive	
Aspirin	40mg p.o. (alternate days), beginning on day 4.
Erythropoietin	2,000 U i.v. x1-2 weekly (if Hct<30),
Anti-CMV and/or antibiotic prophylaxis when considered necessary	

Some immunosuppressive regimens that have proven moderately successful in pig kidney transplantation in NHPs (**Table 5**) may be considered too intensive for the treatment of diabetic patients receiving a pig islet transplant. A less intensive regimen may need to be developed. Park and his colleagues in South Korea succeeded in rendering diabetic monkeys insulin-independent for approximately 2 years following transplantion of adult WT pig islets using an immunosuppression protocol including anti-CD154mAb (38). When this group substituted anti-CD154mAb treatment with an anti-CD40mAb (58), they were unable to replicate these exceptional findings. Moreover, Park et al. demonstrated that a second islet infusion successfully restored normoglycemia under a clinically applicable maintenance immunosuppressive regimen, without the need for further induction therapy (61). Other co-stimulation-blockade agents, such as CTLA4-Ig, have been less efficient in protecting a xenograft (62). The use of islet transplantation from multi-transgenic pigs combined with anti-CD154 mAb-based therapy seem a promising avenue for successful engraftment.

In summary, genetic modifications in porcine islets aim to enhance insulin production and functionality but introduce complexities such as potential immunogenicity and alterations in islet physiology, impacting their viability and function. Addressing these concerns necessitates precision in gene-editing to minimize unintended effects, thorough preclinical evaluations for safety and efficacy, and adherence to ethical standards in genetic engineering. These measures are critical for advancing porcine islet xenotransplantation as a viable treatment option for diabetes, ensuring both the effectiveness and safety of genetically modified islets.

## The problem and prevention of IBMIR

One of the main difficulties in porcine islet xenotransplantation is the initial inflammatory and immune reaction to the transplant – IBMIR (28–30, 32, 63, 64).

**Figure 6:**
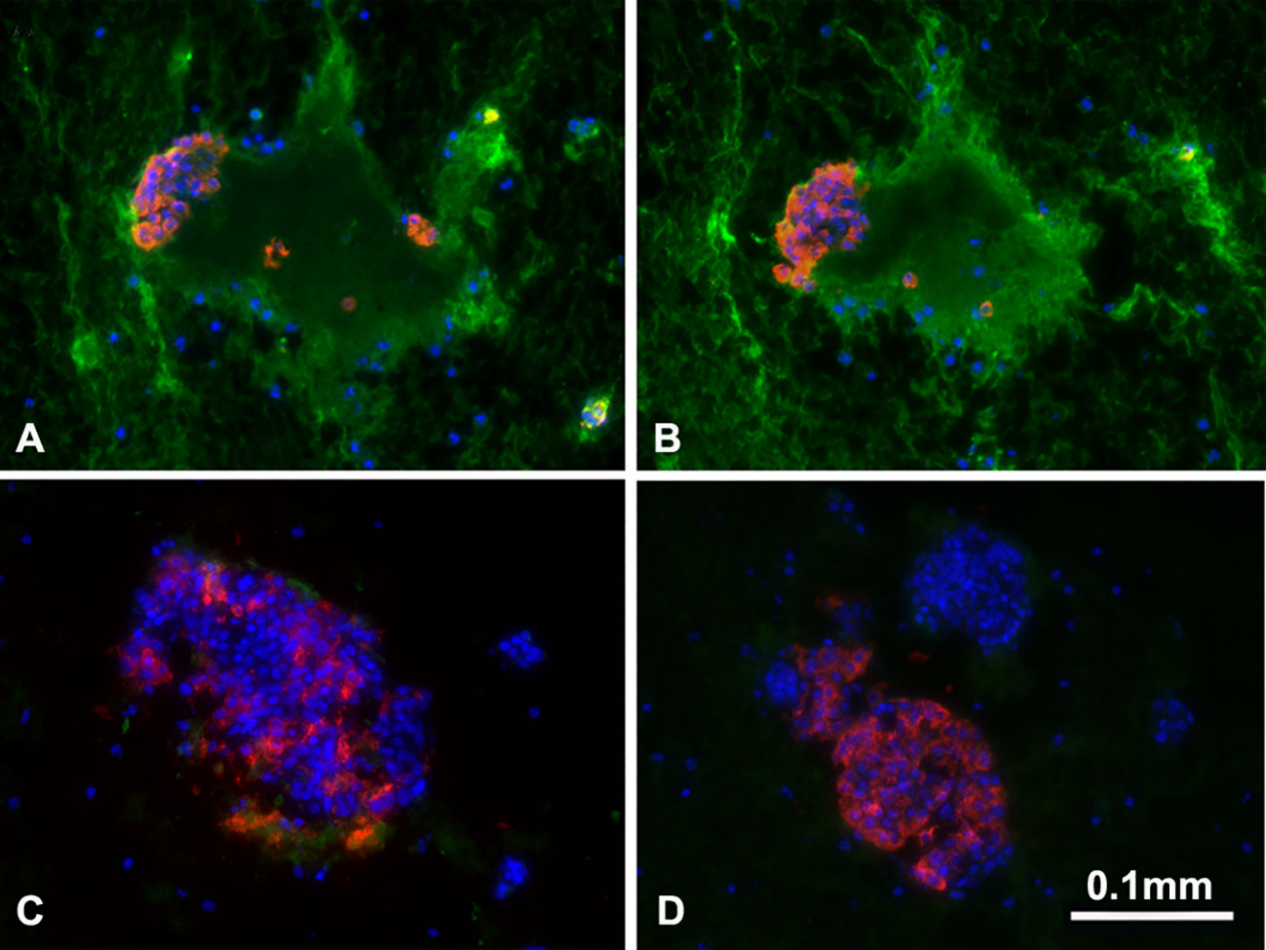
Binding of human IgM and IgG antibody to pig islets (xenogeneic) **(A, B)** and to human islets (allogeneic) **(C, D)**. IgM (green, **A, C**), IgG (green, **B, D**), insulin (red), nucleus (DAPI/blue). Yellow indicates colocalization of insulin and IgM/IgG. The greatly increased binding of human IgM and IgG to pig islets (compared to human islets) is obvious. (Reproduced with permission from 63).

IBMIR occurs when pig islets are introduced into the portal vein, which is currently the preferred location for allotransplantation. When blood comes into contact with islets, especially xenogeneic islets, it triggers an inflammatory response that activates the complement and coagulation systems. As a result, the islets are quickly destroyed. One of the triggers of IBMIR is the expression of tissue factor on the islets (31, 65, 66), as well as the activation of complement and coagulation (63, 64). In addition, the binding of the host’s natural anti-pig antibodies to the islets further exacerbate IBMIR-mediated damage (**Figure 6**). In line with these mechanistic observations, various complement inhibitors and anti-inflammatory agents have been demonstrated to modulate early islet loss (29, 31, 32), e.g., heparin, thrombin inhibitors, and anti-platelet agents (28, 31, 67, 68).

**Figure 7:**
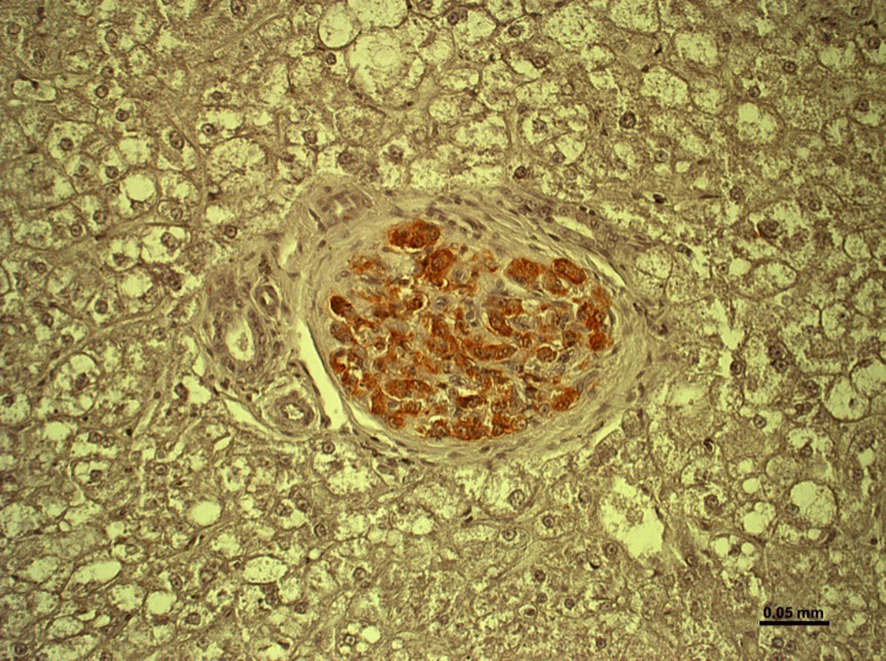
Healthy islet in the liver of an immunosuppressed cynomolgus monkey 12 months after hCD46-transgenic pig islet transplantation. (Reproduced with permission from 69).

Targeting IBMIR and immune rejection seems equally important to ensure that pig islet grafts survive and function in the liver (**Figure 7**) (69).

The destruction of pig islet grafts by IBMIR and rapid antibody-mediated rejection are events that have similarities and differences, but they share common features (63). Many of the genetic modifications that influence IBMIR have a significant impact on reducing antibody-induced rejection (5). Downregulation of pig antigen expression, as well as transgenic expression of human complement- and coagulation-regulatory proteins have all been shown to protect organ and islet grafts (44, 70, 71). With relevance to clinical application, the genetic modifications described do not appear to impair beta cell function *in vivo* —orand *in vitro* (72, 73).

Composite transplantation of porcine islets with mesenchymal stem cells or Sertoli cells demonstrated improved islet engraftment after xenotransplantation (74–81). The mechanisms behind improved islet function are thought to be associated with the anti-inflammatory, regenerative, and immunomodulatory properties of mesenchymal stem cells and Sertoli cells.

## Encapsulation

An alternative approach to protect islets from the recipient microenvironment is to physically isolate the islets by ‘encapsulation. Ongoing investigations propose micro- and macro-structures that isolate the islet grafts from the host immune system, while also ensuring the provision of oxygen and nutrients to the enclosed cells and tissues (79, 82–86). Encapsulation technology in islet xenotransplantation offers the theoretical advantage of immunoprotection, potentially eliminating the need for systemic immunosuppression. It aims to create a semi-permeable barrier that shields transplanted islets from immune cells while allowing insulin, nutrients, and oxygen to pass through. However, this approach faces challenges, including the risk that the biomaterials may permit cytokine penetration, potentially triggering an immune response, and the possible insufficiency of oxygen and nutrient transport across the encapsulation barrier, which could lead to islet dysfunction or loss. These limitations underscore the need for ongoing research to optimize encapsulation materials and techniques for successful xenotransplantation outcomes.

## Sensitization to HLA or to pig xenoantigens

Two important questions have arisen. The first Is whether sensitization to human leukocyte antigens (HLA) harms pig islet xenotransplantation. Blood transfusions, human organ transplants, or pregnancies can trigger the generation of antibodies directed towards HLA antigens. In these instances, if an organ or cell transplant is required, preexisting anti-HLA antibodies can pose challenges in finding a suitable human donor for organ or cell transplantation. There is evidence that anti-HLA antibodies may target some swine leukocyte antigens (SLA), due to cross-reactivity, but cross reactivity is expected to be minimal, thus unlikely negatively affecting xenotransplantation (reviewed in 87).

The second question is whether sensitization to SLA would be detrimental to subsequent human islet allotransplantation. If sensitization to a pig xenograft develops, the existing limited information suggests that the recipient would not be at an immunological disadvantage to subsequently undergo allotransplantation (reviewed in 87).

## The induction of immune tolerance

The ultimate goal of organ and cell allo- or xeno-transplantation is to induce a state in which the host immune system recognizes the transplanted pig islets as ‘self’ and makes no effort to reject them. Discontinuing all immunosuppressive therapy would be possible if immunologic ‘tolerance’ could be attained. Immune tolerance to allografts has been explored by different approaches, e.g., (i) donor-specific hematopoietic progenitor cell transplantation (chimerism) or (ii) concomitant donor-specific thymus transplantation (88). The role of regulatory cells, however, in immune tolerance remains uncertain (89).

In contrast to allotransplantation with deceased donor organs, xenotransplantation offers the advantage of elective timing of the transplant, which provide a time window for the manipulation of the host’s immune system towards immune tolerance. In light of this potential advantage, if the early inflammatory events causing IBMIR, can be successfully modulated, immune tolerance might be achievable to control cellular rejection.

## Improving function of porcine islets

Compared to humans, the porcine islet response to stimuli presents some differences requiring further investigation. Pigs use less insulin, need lower levels of C-peptide, and sustain higher blood glucose levels in comparison to NHPs (**Table 6**) (72, 73, 92). When stimulated with glucose *in vitro*, isolated porcine islets secrete 3 to 6 times less insulin than human islets (86, 93, 94). Genetic modifications aimed at enhancing islet function and insulin production in pig islets have been explored (95, 96). However, there is some concern that forcing insulin secretion might result in islet metabolic imbalance, and ‘exhaustion’ (86). To overcome this potential problem, transplantation of a greater number of islets may provide a solution.

**Table 6:** Fasting blood glucose, C-peptide, insulin, and glucagon levels in cynomolgus monkeys (*Macaca fascicularis*), pigs, and humans *^a^*.

	Cynomolgus monkeys^222a^	Pigs^222a^	Humans
**Blood glucose (mmol·L^−1^)**	2.2 – 4.1 (3.2)	4.0 – 5.2 (4.8)	3.9 – 5.6^222b^
**C-peptide (nmol·L^−1^)**	0.47 – 3.14 (1.39)	0.11 – 0.32 (0.16)	0.17 – 0.66^222c^
**Insulin (pmol·L^−1^)**	15 – 201 (109)	7 – 12 (9)	34 – 138^222c^
**Glucagon (pmol·L^−1^)**	18.7 – 179.4 (54.3)	11.3 – 13.8 (12.5)	5.7 – 28.7^222c^

Data are presented as ranges (mean). C-peptide (p<0.001), insulin (p=0.021) and glucagon (p<0.001) levels were significantly higher in monkeys than in pigs, while blood glucose levels were significantly (p<0.001) lower in monkeys. Human data are obtained from the literature and were measured in venous plasma (90, 91).

^a^Table based on Casu et al. (92).

## Metabolic aspects and glucose ‘counter-regulation’

The ability to control blood glucose levels within a normal range is dependent on the interaction of several factors. Endocrine hormones of the pancreas, paracrine effects, the release of neurotransmitters and neuropeptides, gluconeogenesis and glycogenolysis all play roles in maintaining blood glucose levels. These parameters differ between species, thus raising questions on the potential effects of cross-species metabolic variability in the context of xenotransplantation (92). Understanding the metabolic differences between pigs and humans, and the potential ramifications, is vital for the advancement of clinical xenotransplantation.

Parameters of metabolic control are more similar between pigs and humans than between pigs and NHPs (92). However, pigs are more glucose tolerant and have lower basal insulin levels than humans (97). Thus, metabolic control may be more easily established in pig-to-human than in pig-to-NHP islet transplantation. In response to glucose changes, both isolated neonatal and adult porcine islets demonstrate coherent insulin and glucagon secretion and suppression *in vitro*. A high concentration of glucose increases insulin secretion and inhibits glucagon secretion. Alpha cells may play a more prominent role in the response to glucose changes in pigs than in humans. Glucagon secretion is more pronounced in neonatal compared to adult pig islets (98).

Taken together, these data suggest not only that the metabolic profile of porcine islets may be similar to human islets but also that the highly efficient glucagon response to hypoglycemia may represent a clinically relevant factor predictive of timely glucose counter-regulation.

One aspect of pig metabolism that has not yet been fully explored has emerged from a genetic study aimed on the “thrifty gene hypothesis” in human populations (99). According to this hypothesis, humans have survived famine and starvation for millennia, thus certain populations may have genes that determine increased fat storage, which would facilitate survival in times of want or famine. Nonetheless, in an environment characterized by easy access to food, as in modern Western cultures, for example, such genes predispose the genetic carrier to develop type 2 diabetes. In contrast to this outlook for humans, domesticated pigs and cows have long been selectively bred for their ability to efficiently accumulate and store energy (for later consumption by humans). Pigs and cows should, therefore, be protected against the toxic effects of a “diabetogenic” environment (i.e., one that favors inactivity and energy abundance).

The mechanisms that determine this resistance to diabetes are not fully understood. However, it is known that pigs do not accumulate amyloids (100) and are, therefore, resistant to amyloidosis, which is one of the pathological hallmarks of diabetes (101). Porcine islets transplanted into mice do not accumulate amyloids, in contrast to human islets (100). Similar observations were reported when porcine islets were transplanted into NHPs (44). Sequencing of porcine islet amyloid polypeptide (IAPP, or amylin, the peptide responsible for formation of fibrils of amyloids) and comparison with human IAPP demonstrated 10 substitutions that differentiated the porcine form from the human form and contributed to reduced amyloidogenesis. Reduced toxicity of porcine IAPP was, indeed, demonstrated *in vitro* in rat (INS) cells (100).

Moreover, genetic engineering of pig donor tissues, including the introduction of human transgenes expressed under an insulin promotor, do not appear to affect glucose metabolism (72, 73, 102).

## Clinical trials of pig islet xenotransplantation

With the exception of studies by Groth et al. (13), and Wang et al. (16, 103), free islet xenotransplantation has not undergone clinical testing, though there have been several clinical experiments or trials involving encapsulated islets in the absence of immunosuppressive therapy (6). None has been totally successful. In some of these experiments it was unclear whether improved glycemic control was associated with meticulous medical management (i.e., attention to diet, glucose monitoring, and expert medical attention) rather than to insulin production by the pig islets. However, Matsumoto et al. demonstrated a substantial reduction of HbA1c levels for >600 days in recipients of encapsulated porcine islets in the absence of immunosuppressive therapy (17, 18). Minimal adverse events were reported, but improved and more consistent efficacy is still required.

Islet-source pigs will be housed in biosecure ‘designated pathogen-free’ facilities that eliminate most potentially-pathogenic microorganisms. By implementing Good Manufacturing Practices and established Standard Operating Procedures, the risk of transfer of a pathogenic microorganism is considered small (104–106). Although there were initial worries that porcine endogenous retroviruses (PERV) could become activated in humans, the risk, although hitherto unknown, is also thought to be small (14, 107, 108). Furthermore, if necessary, PERV-KO is possible (37, 109, 110).

Because the risk to the recipient is considered to be low, clinical trials of pig islet transplantation should possibly not be held to the high standards expected of pig organ xenotransplantation. This particularly relates to trials of encapsulated islets in which *no* immunosuppressive therapy is administered (105, 106).

According to the regulations of the U.S. Food and Drug Administration (FDA), it is required to prioritize the selection of patients who (i) suffer from a life-threatening disease with no access to effective alternative treatment, and (ii) have the potential to experience a noteworthy enhancement in their quality of life following the procedure (111). Individuals suffering from diabetes who are facing repeated and intense unawareness of hypoglycemia even after receiving the best possible medical treatment may be the most appropriate individuals to consider as potential candidates. Those with diabetic nephropathy would benefit from the successful transplantation of both a pig kidney and pig islets. The low risk in pig islet xenotransplantation trials is attributed to rigorous safety protocols, including genetic engineering of pigs to reduce human immune reactions and meticulous screening for pathogens. This approach minimizes potential zoonotic infections and immunogenic complications. Compliance with FDA regulations is ensured through adherence to established guidelines for xenotransplantation, encompassing product safety, ethical standards, and clinical trial conduct. Detailing these aspects can enhance the research’s credibility, demonstrating a commitment to safety, regulatory compliance, and ethical considerations in advancing xenotransplantation as a therapeutic option. Furthermore, if the islets are rejected, this is unlikely to be life-threatening for the patient.

## Potential insights from single-cell RNA sequencing

The advent of scRNA-seq has inaugurated a new era in the molecular dissection of biological processes. This technique, distinguished by its capacity to unravel the complexities of gene expression at an individual cell level, may prove pivotal in demystifying the heterogeneity inherent within cellular populations (112). This granularity of data may prove valuable in elucidating the nuanced interplays that govern both physiological and pathological states in complex biological systems.

In the realm of xenotransplantation, scRNA-seq may facilitate resolution in characterizing diverse cell types within a xenograft, encompassing the spectrum from immune cells to specialized graft cells (113). This advanced molecular profiling may afford insight into the intricacies of immune rejection mechanisms, graft tolerance phenomena, and the overarching molecular orchestration of transplantation (114). The ability of scRNA-seq to pinpoint cellular stress responses and pathophysiological transformations within xenografts may help refine transplantation strategies and prolong graft viability.

In the specific context of islet transplantation, scRNA-seq has already begun to demonstrate its potential. By dissecting the molecular heterogeneity of islet cells and delineating the complex immune interactions post-transplantation, scRNA-seq may help reshape our comprehension of graft dynamics (115). This molecular clarity may optimize immunomodulatory approaches post-transplantation and enhance overall graft efficacy.

The integration of scRNA-seq into pig islet xenotransplantation research may not only improve our understanding of transplanted islet cell biology but also pioneer novel therapeutic avenues for Type 1 diabetes.

## Comment and conclusions

We anticipate that eventually pig free islet transplantation will offer a clinically-applicable therapy for patients with T1D. We suggest that this will be a preferable approach to any form of implantation of encapsulated islets, and that the intensity of immunosuppressive therapy that is required will not be prohibitive.

Porcine islets appear to be metabolically compatible with human islets, with potential advantages in glucose counter-regulation, resistance to beta cell damage, and resistance to a diabetogenic lifestyle.

## Author contributions

DC: Conceptualization, Funding acquisition, Writing – original draft, Writing – review & editing. LM: Writing – original draft, Writing – review & editing, Funding acquisition. RB: Writing – original draft, Writing – review & editing.

## Footnotes

1 Kinoshita,K, Maenaka A, Rosales I, Karadagi A, Tomsugi T, Ayares D, et al. Novel factors potentially initiating acute antibody-mediated rejection in pig kidney xenografts despite an efficient immunosuppressive regimen. Xenotransplantation (In press) (2024).
